# Differential Toxicity of DNA Adducts of Mitomycin C

**DOI:** 10.4061/2010/698960

**Published:** 2010-07-29

**Authors:** Jill Bargonetti, Elise Champeil, Maria Tomasz

**Affiliations:** ^1^Department of Biological Sciences, Hunter College, The City University of New York, New York, NY 10021, USA; ^2^Department of Science, John Jay College, The City University of New York, New York, NY 10019, USA; ^3^Department of Chemistry and Biochemistry, Hunter College, The City University of New York, New York, NY 10021, USA

## Abstract

The clinically used antitumor agent mitomycin C (MC) alkylates DNA upon reductive activation, forming six covalent DNA adducts in this process. This review focuses on differential biological effects of individual adducts in various mammalian cell cultures, observed in the authors' laboratories. Evidence is reviewed that various adducts are capable of inducing different cell death pathways in cancer cells.This evidence is derived from a parallel study of MC and its derivatives 2,7-diaminomitosene (2,7-DAM) which is the main metabolite of MC and forms two mono-adducts with DNA, and decarbamoyl mitomycin C (DMC), which alkylates and cross-links DNA, predominantly with a chirality opposite to that of the DNA adducts of MC. 2,7-DAM is not cytotoxic and does not activate the p53 pathway while MC and DMC are cytotoxic and able to activate the p53 pathway. DMC is more cytotoxic than MC and can also kill p53-deficient cells by inducing degradation of Checkpoint 1 protein, which is not seen with MC treatment of the p53-deficient cells. This difference in the cell death pathways activated by the MC and DMC is attributed to differential signaling by the DNA adducts of DMC. We hypothesize that the different chirality of the adduct-to-DNA linkage has a modulating influence on the choice of pathway.

The mitomycins are a group of highly potent antibiotics, produced by the microorganism *Streptomyces caespitosus*, as discovered in Japan in the 1950s [1]. The prototype, most studied member of this group is mitomycin C (**1;** MC) (Figure 1). On account of its broad-spectrum antitumor activity, MC has been widely used in clinical cancer chemotherapy [2].

 MC has been recognized as a classical DNA damaging agent, on account of its monofunctional and bifunctional DNA alkylating activity. Early studies have revealed an extraordinary property of the mitomycins; they were found to cross-link the complementary strands of DNA *in vivo* and *in vitro* [3]. MC was postulated to have two alkylating centers, the 1,2-aziridine and 10-carbamate groups (Figure 1). The mitomycins were the first natural antibiotics found possessing DNA cross-linking activity; there has been only one other natural cross-linker discovered since, carzinophyllin/azinomycin [4]. Synthetic DNA cross-linkers have become a paradigm of anticancer treatment, however. 

 Our laboratory has elucidated the structure of the DNA cross-link adduct of MC [5] and five additional monofunctional DNA adducts formed in MC-treated tumor cells as well as their DNA sequence selectivities, as summarized in several reviews (see, [6] and references therein). The structures of the six MC adducts are shown in Figure 2. 

 Numerous synthetic and natural analogs of the mitomycins have been discovered and studied; the details of which are beyond the scope of this review. However, two closely related derivatives of MC and their DNA adducts are relevant to the present subject, namely, 2,7-diaminomitosene (**2**; 2,7-DAM) and 10-decarbamoyl mitomycin C (**3;** DMC) (Figure 1). Their DNA adducts are described in the context of this review as follows.

 2,7-DAM is a major metabolite isolated from cells and tissues treated with MC. It is a byproduct of the reductive activation of MC required for unmasking MC's alkylating activity [7, 8]. Our laboratory found that two of the six DNA adducts formed in MC-treated cells are derived from monofunctional activation of DNA by the nascent metabolite of MC, 2,7-DAM [9, 10]. The structures of the two adducts were determined (**5** and **6**; Figure 2) [10, 11]. When tumor cells were treated with 2,7-DAM, the two adducts **5** and **6** were formed at high frequency [10]. Surprisingly, 2,7-DAM was not cytotoxic under aerobic conditions and negligibly cytotoxic under hypoxic treatment relative to MC [12, 13]. Its adduct **5** was specifically shown to be noncytotoxic and nonmutagenic in *E. coli* and simian kidney cells [14]. Consistent with these findings, in cell-free systems template oligonucleotides containing a single 2,7-DAM-dG-N7 adduct directed selective incorporation of cytosine opposite to this adduct in primer strands, catalyzed by Klenow (exo-) DNA polymerase [14]. Although no analogous experiments were performed with 2,7-DAM adduct **6** the nontoxicity and nonmutagenicity of the parent drug [14] predict an outcome similar to that of **5**. 

 Proof for the cross-link adduct as the critical cytotoxic lesion among the six MC adducts was provided by a study of 10-decarbamoyl mitomycin C (**2; **DMC), an artificial derivative obtained from MC by chemical removal of the 10-carbamoyl group [15]. DMC has long been regarded as an innocuous monofunctional MC derivative, incapable of causing ICLs, on account of lacking its carbamate alkylating center. Accordingly, in two laboratories, incubation of purified DNA and chemically activated DMC yielded only a monofunctional adduct **2a** as the major product, with no evident DNA cross-link adduct detectable [16, 17]. Inconsistent with these findings, however, DMC was reported to be slightly more cytotoxic than MC to hypoxic EMT6 mouse mammary tumor cells as well as to CHO cells [18, 19]. To resolve this paradox, EMT6 cells were treated with MC or DMC under hypoxia at equimolar concentrations and the resulting DNA adducts were determined structurally and quantitatively [20]. DMC treatment generated two stereoisomeric monoadducts **2a **and **2b **(1′′-alfa and 1′′-beta, resp.) and two ICL adducts, similarly stereoisomeric at the 1′′ position **3a **and** 3b **(1′′-alfa- and 1′′-beta isomers, resp.) (Figure 3). In addition, the cytotoxicities of MC, DMC, and 2,7-DAM were determined [20], confirming the earlier results described above. Overall, the adduct frequencies with DMC were much higher (20- to 30-fold) than with MC. Although DMC monoadducts greatly exceeded DMC ICL adducts (approximately 10 : 1 ratio), the latter were equal or somewhat higher in number than the ICL adducts from MC. The relatively similar cytotoxicities of MC and DMC correlated with the relatively similar ICL adduct frequencies of the two drugs, but not with their relative monoadduct or total adduct frequencies. This correlation may be regarded as specific experimental evidence that in the EMT6 tumor cell line ICLs rather than monoadducts are the critical factors in the cell death induced by MC [20].

 It is important to note, however, that in sharp contrast to the two 2,7-DAM monoadducts **5 **and **6**, the MC monoadduct **1a **and MC and DMC common monoadduct **2a** were shown to be highly inhibitory to phage replication after transfection in *E. coli* [21] and strong blocks of DNA synthesis in cell-free systems [22]. Furthermore, monoadduct **1a **was shown to be nonmutagenic in *E. coli*. The biological experiments utilized transfection of single-site adducted M13 phage or synthetic oligonucleotide constructs in *E. coli* strains [22].

 Isolation and quantitative analysis of DNA adducts of MC, DMC, and 2,7-DAM formed in tumor cells have been conducted by the Tomasz lab in collaboration with the Sartorelli-Rockwell group at Yale University, using almost exclusively EMT6 mouse mammary tumor cells. Recently, the number of cell lines was expanded in collaboration with the Bargonetti group, and a new method of quantitative analysis of the adducts of MC and DMC was employed, namely, LC/electrospray tandem mass spectrometry [23]. In this work Fanconi Anemia-A cells, normal human fibroblasts, MCF-7 human breast cancer cells, and EMT6 mouse mammary tumor cells were treated with MC or DMC under identical conditions, then the cellular DNA was isolated and analyzed for adducts. In addition to the previously known 1′′-alfa isomer adducts of MC the analysis included the new 1′′-beta stereoisomers from DMC. The results confirmed the generality of the DMC adduct pattern described above for EMT6 cells [20] in each cell line (Figure 4). It was concluded that DMC shows a stereochemical preference of linkage to the guanine-2-amino group *opposite* from that of MC; it forms two stereoisomeric ICL adducts, and its monoadducts are formed overall at much greater frequencies than its ICLs. The adduct patterns of the Fanconi Anemia-A cells and the control healthy fibroblast cells were identical. This work also provided preliminary evidence for differential removal of adducts upon post-treatment incubation of the cells in drug-free media. However, more work is needed to confirm these preliminary results.

In response to DNA adducts mammalian cells activate a complex signal transduction cascade to activate cell cycle checkpoints, initiate DNA repair, or induce cell death. The Bargonetti group, in collaboration with the Tomasz group, has investigated the biological signaling of mitomycin DNA-adducts using a human tissue culture model [13, 24]. The studies addressed two key areas: the ability of 2,7-DAM, MC, and DMC to activate the p53 pathway, and the differing ability of the drugs to induce cell death in either p53-proficient or p53-deficient cells. The p53 protein is a central player in the mammalian cellular response to DNA damage [25]. Following DNA damage the p53 protein is stabilized and functions as a strong transcriptional activator of genes that are required for cell cycle checkpoints, DNA repair, and the induction of cell death [26]. Many cancer cells have lost this response through sporadic mutations that occur in the p53 gene and thus the cells become deficient in the p53-pathway. Over 50 percent of all cancers have sustained mutations to the p53 DNA binding domain and for the purposes of this review we will call them p53-deficient. MC and DMC treatment of mammalian cells that are p53-proficient results in increased p53 protein, activation of the p53-mediated transcriptional activation of p53-pathway genes, and apoptotic cell death [13, 24], while the noncytotoxic mitomycin derivative DAM does not activate the p53 protein or the p53 pathway [13]. This is in strong keeping with the paradigm of DNA damage-mediated cytotoxicity being associated with the activation of the p53 pathway in p53-proficient cells. DNA damage initiates the activation of DNA repair-related kinases ATR, ATM, Chk1, and Chk2 [27]. Both ATM and ATR kinases are able to phosphorylate and activate p53. The fact that 2,7-DAM does not cause activation of the p53-pathway strongly suggests that the noncytotoxic monoadducts that result following 2,7-DAM treatment (**5 **and **6**) are unable to activate the DNA damage-associated kinase pathways. The comparison of the ability of 2,7-DAM, MC and DMC to activate the p53-pathway demonstrates that 2,7-DAM monoadducts do not activate this critical checkpoint pathway while MC- and DMC-mediated crosslinks are able to do so. DNA interstrand crosslinks (ICLs) are able to use ATR/Chk1 and the Fanconia anemia pathway to signal for cellular checkpoints [28]. Therefore, the 2,7-DAM monoadducts are likely to be repaired using a different pathway.

The ability of mitomycin DNA adducts to sensitize p53-deficient cancer cells to die has been examined by a comparison of the influence of MC and DMC on human cancer cells. Cancer cells that are p53-deficient rely on their ATR/Chk1 checkpoint as their last means of survival and if they lose Chk1 they become more sensitive to death [29, 30]. However, DNA damage can also cause strong activation of ATR that subsequently results in activation of Chk1 ubiquitination and targeted proteolysis of the protein [31]. The loss of Chk1 in p53-deficient cells causes the cells to lose their last remaining cell cycle checkpoint and the cells then die by a caspase 2-mediated pathway [32]. In p53-deficient cells DMC is more cytotoxic than MC [13, 24] and this associates with a reduction in Chk1 upon DMC but not MC treatment [24]. Recent work from the Bargonetti and Tomasz groups suggests that increased cytotoxicity of DMC relative to that of MC might be due to the variable chirality of the DMC- DNA adducts that activate Chk1-mediated proteolysis [23, 33]. Interstrand crosslinks activate the ATR/Chk1 pathway as part of their signal transduction response [28] but variable chirality has yet to be proven to differentially modulate such a pathway. However, it has been shown that Fanconi anemia proteins and excision repair proteins participate in recognition of MC DNA adducts [34]. It will be interesting to determine if altered chirality of the mitomycin ICL adducts differentially recruit the DNA damage signaling machinery to the DNA. This could be the consequence of a difference in the alignment of the 1′′-alfa and 1′′-beta stereoisomeric adducts in DNA. Structural studies in coordination with biochemical techniques to examine specific DNA-protein interactions could further explore this hypothesis. 

*In summary;* using a variety of techniques it has been possible to assign specific biological effects to each of the six DNA adducts of MC in mammalian cell cultures. Critical in this endeavor was the surprising discovery that DMC was slightly more cytotoxic than MC in cell cultures and it produced ICLs which correlated with its cytotoxicity [20]. This served as proof that the ICL was the major cytotoxic lesion of MC and DMC. DMC formed 20–30 times more DNA monoadducts than MC, which were linked to the N^2^ atom of guanines with opposite chirality (I′′-beta) to that of MC (1′′-alfa) [23]. Parallel studies of MC and DMC revealed that DMC is much more cytotoxic to p53-deficient cells than MC [13, 24, 33] and we have shown that it is capable of inducing a cell death pathway involving proteasome-mediated degradation of Checkpoint 1 protein [33]. Future studies in our laboratories aim at elucidating the mechanism of the differential cell death pathways of MC and DMC in the context of the structures of their DNA adducts. More than 50% of human cancers are deficient for p53 function and contain either p53 mutations or oncogenic proteins that functionally inactivate p53. Determining how alternative DNA-adduct chirality can initiate signal transduction pathways for increased cytotoxicity in p53-deficient cancers could become a new paradigm in chemotherapeutic drug discovery.

## Figures and Tables

**Figure 1:**
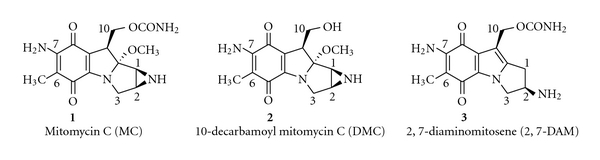
Chemical structures of MC, DMC, and 2,7-DAM. (Reproduced with permission from *Chem. Res. Toxicol.* **2008,** *21*, 2370).

**Figure 2:**
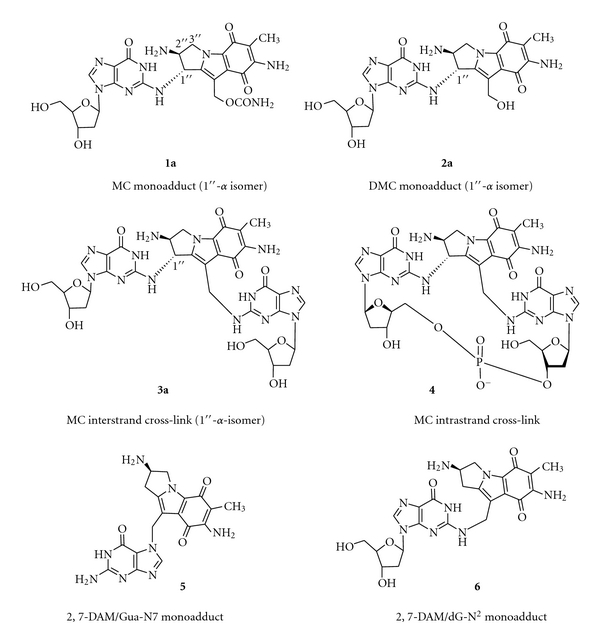
Chemical structure of six major adducts of reductively activated MC. (Reproduced with permission from the reference in Figure 1.

**Figure 3:**
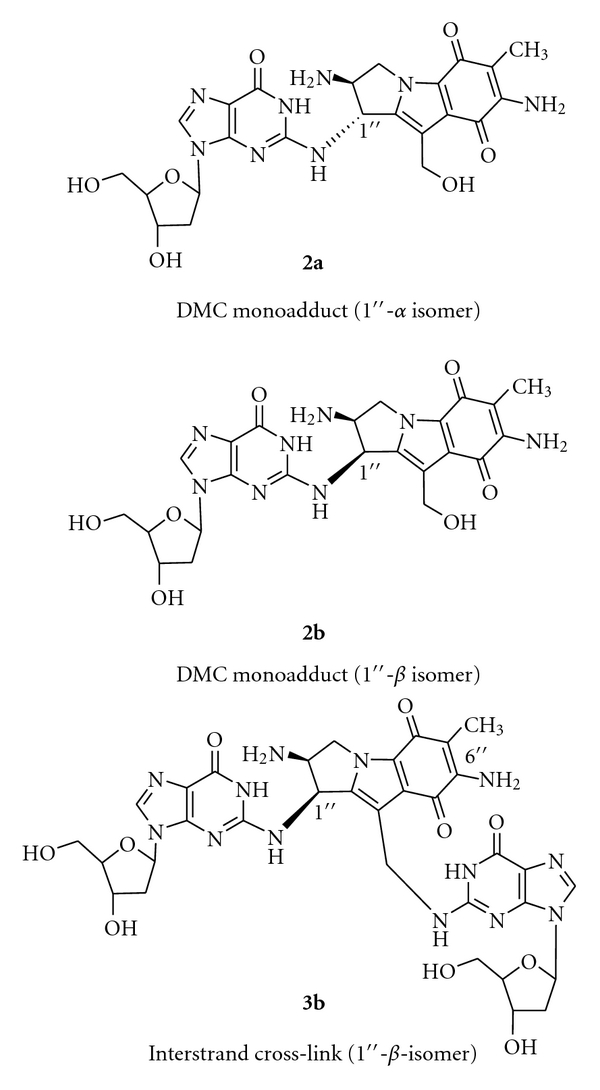
Chemical structures of major adducts of DMC. (Reproduced with permission from the reference in Figure 1.

**Figure 4:**
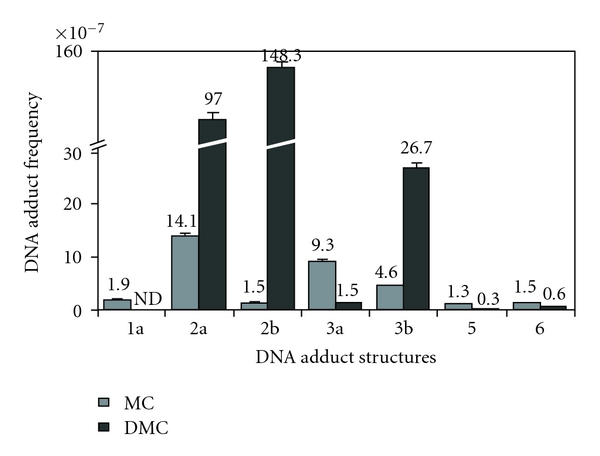
Frequencies of DNA adducts formed in MCF-7 human breast cancer cells treated with 10 *μ*M MC (gray bars) or 10 *μ*M DMC (black bars) for 24 hours under aerobic conditions. The individual frequency values of the bars are indicated above the bar error limits. Error limits are shown in % relative standard deviation units. (Reproduced with permission from the reference In Figure 1.
